# Lung cancer-derived galectin-1 contributes to cancer associated fibroblast-mediated cancer progression and immune suppression through TDO2/kynurenine axis

**DOI:** 10.18632/oncotarget.8488

**Published:** 2016-03-30

**Authors:** Ya-Ling Hsu, Jen-Yu Hung, Shin-Yi Chiang, Shu-Fang Jian, Cheng-Ying Wu, Yi-Shiuan Lin, Ying-Ming Tsai, Shah-Hwa Chou, Ming-Ju Tsai, Po-Lin Kuo

**Affiliations:** ^1^ Graduate Institute of Medicine, College of Medicine, Kaohsiung Medical University, Kaohsiung, Taiwan; ^2^ Division of Pulmonary and Critical Care Medicine, Kaohsiung Medical University Hospital, Kaohsiung, Taiwan; ^3^ School of Medicine, College of Medicine, Kaohsiung Medical University, Kaohsiung, Taiwan; ^4^ Institute of Clinical Medicine, College of Medicine, Kaohsiung Medical University, Kaohsiung, Taiwan; ^5^ Division of Chest Surgery, Department of Surgery, Kaohsiung Medical University Hospital, Kaohsiung, Taiwan; ^6^ Institute of Medical Science and Technology, National Sun Yat-Sen University, Kaohsiung, Taiwan

**Keywords:** galectin-1, kynurenine, lung cancer, cancer-associated fibroblasts

## Abstract

Communication between cancer cells and their microenvironment plays an important role in cancer development, but the precise mechanisms by which cancer-associated fibroblasts (CAF) impact anti-cancer immunity and cancer progression in lung cancer are poorly understood. Here, we report that lung fibroblasts when activated by lung cancer cells produce tryptophan metabolite kynurenine (Kyn) that inhibits dendritic cells' differentiation and induces cancer growth as well as migration. We identified TDO2 (tryptophan 2,3-dioxygenase) as the main enzyme expressed in fibroblasts capable of tryptophan metabolism. Mechanistically, condition medium of CAF or exogenous kynurenine stimulated AKT, with no lysine 1 (WNK1) and cAMP response element-bindingprotein (CREB) phosphorylation in lung cancer cells. Inhibition of the AKT/CREB pathway prevents cancer proliferation, while inhibition of the AKT/ WNK1 reverted epithelial-to-mesenchymal transition and cancer migration induced by kynurenine. Moreover, we also demonstrate that lung cancer-derived galectin-1 contributes to the upregulation of TDO2 in CAF through an AKT-dependent pathway. Immunohistochemical analysis of lung cancer surgical specimens revealed increased TDO2 expression in the fibroblasts adjacent to the cancer. Furthermore, in vivo studies showed that administration of TDO2 inhibitor significantly improves DCs function and T cell response, and decreases tumor metastasis in mice. Taken together, our data identify the feedback loop, consisting of cancer-derived galectin-1 and CAF-producing kynurenine, that sustains lung cancer progression. These findings suggest that targeting this pathway may be a promising therapeutic strategy.

## INTRODUCTION

Lung cancer is among the most frequent cause of death worldwide, accounting for approximately 1.8 million new cases and 1.6 million deaths in 2012 [1]. Its five-year survival rate is 11%, which is significantly lower than that of other cancers, including colon, breast, and prostate cancer [2]. Complex networks between cancer cells and tumor microenvironments (TME), which are composed of various cell types, extracellular matrices, and metabolic substrates, influence tumor initiation, progression and metastasis. Cancer associated fibroblasts (CAFs), the most abundant “non-cancerous” cells, are considered one of the most important cells that interact with cancer. They are responsible for the creation of a tumor-permissive microenvironment, by increasing tumor angiogenesis and decreasing anti-cancer immunity and tumor angiogenesis, thus representing an attractive target for cancer treatment due to their genetic stability [3]. Target CAF increases antitumor responses of dendritic cell (DC)-based vaccine by decreasing the infiltration of regulatory T cells and myeloid-derived suppressor cells [4]. However, it remains unclear how these CAFs influence anti-cancer immune response.

Metabolic reprogramming is a hallmark of cancer and considered to be critical to supporting accelerated cell proliferation, progression and metastasis [5, 6]. Growing evidence shows that an altered metabolism is also found in TME cells, and that the metabolic coupling between stroma and cancer cells contributes to tumor survival and progression [6]. Imbalances in tryptophan (Trp) metabolism are found in several cancers, and the metabolite kynurenine (Kyn) is recognized as a critical microenvironmental factor that inhibits anticancer immunity by decreasing cell proliferation or inducing apoptosis in T cells [7, 8]. Tryptophan is catabolized by the rate-limiting enzyme indoleamine-2,3-dioxygenase (IDO) or tryptophan-2,3-dioxygenase (TDO) expressed in cancer or immune cells. High Kyn levels or Kyn/Trp ratios in the serum of patients is associated with elevated cancer progression and poor prognosis [9–11]. However, the sources of Kyn in lung cancer patients remain largely unknown, as does Kyn's role as a mediator of CAF-mediated immunosuppression. Understanding the multidirectional interaction of CAF, cancer, and immune cells could be great benefit in developing a promising TME-targeted strategy against cancer.

Galectins are a family of mammalian β-galactoside-binding proteins that have been to be associated with immune suppression and cancer development [12, 13]. Growing evidences indicated that the galectin-3 is involved in regulating the tumor development by inhibiting host immunity [12]. Our previously reported that galectin-1, serves as a specific cancer-derived factor to inhibit DC differentiation and function in lung cancer [14]. Inhibition of galectin-1 in CAF decreases the expression of monocyte chemotactic protein-1 (MCP-1/CCL2), which has been implicated in contributing to an immunosuppressive TME, and as an inducer of cancer progression [15, 16]. Here we investigate whether CAF is responsible for immunosuppression of TME by impairing DC differentiation and function in lung cancer. The inhibitory effect of CAF is mediated by elevated levels of Kyn, which is triggered by lung cancer-derived galectin-1. We also report that a CAF-targeted strategy restores antitumor immune responses and reduces lung cancer metastasis.

## RESULTS

### Lung cancer associated fibroblast (LCAF) impairs DC differentiation and function

To establish the study of LCAF, we co-cultured primary normal human lung fibroblasts (NHLF) with human lung cancer cell lines CL1-5 and A549. As shown in Supplementary Figure S1A, co-culture NHLF with lung cancer cells increased the expression of CAF marker α-smooth muscle actin (α-SMA). In addition, the CMs of CL1-5 and A549 cells also enhanced the expression of α-SMA in NHLF (Supplementary Figure S1B), suggesting that lung cancer switch lung fibroblast to myofibroblast (LCAF).

**Figure 1:**
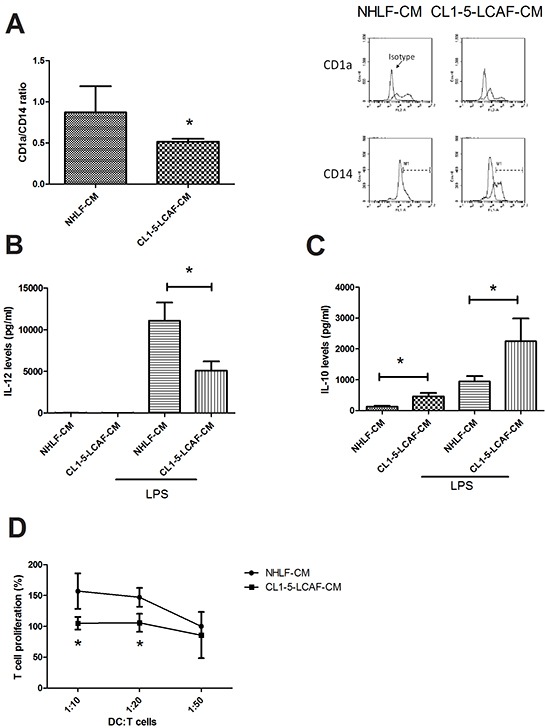
LCAF impedes the differentiation and function of DCs **A.** The CMs of LCAF decreased the differentiation of DCs from CD14^+^ monocytes. The CMs of LCAF decreased IL-12 **B.** and increased the IL-10 **C.** in DCs. Reduced function of LCAF-derived DCs on T cell proliferation **D.** Normal human lung primary fibroblasts (NHLF) were cultured with or without CL1-5 cells for 24 h, and the condition medium was collected (NHLF-CM and CL1-5-LCAF-CM). CD14^+^ monocytes were cultured with various CMs (20%) containing GM-CSF (20 ng/ml) and IL-4 (20 ng/ml) for 5 days. After stimulation by IFN and LPS, mature DCs were co-cultured with naïve CD4 T cells for another 5 days. The expression of DCs' surface markers was measured by flow cytometry, and the levels of cytokines in the DC supernatants were determined by MILLIPLEX MAP kits. All results are representative of at least three independent experiments, and each value is the mean ± SD of three determinations. The results were reported as mean ± SD; *p < 0.05.

To investigate whether LCAF contributes to tumor immunity evasion, we assessed the effect of LCAF on DCs' differentiation and function. Our study shows that lung cancer cells impair the differentiation of DCs, supported by decreasing CD1a up-regulation and the disappearance of CD14 [14]. Therefore, the ratio of CD1a/CD14 is a critical indicator for assessing the differentiation degree of DCs in lung cancer. Figure 1A shows that CL1-5-LCAF-CM impairs the differentiation of DCs from CD14+ monocytes, compared to NHLF-CM. In addition, CL1-5-LCAF-CM also decreased the ability of DCs to produce proinflammatory cytokine IL-12 after LPS stimulation, whereas it increased secretion of immunosuppressive IL-10 regardless of LPS treatment (Figure 1B and 1C). The major function of DCs is to present antigens to the T cells and priming T response. In comparison with those cultured in NLHF-CM, CL1-5-LCAF-CM conditioned DCs showed impaired ability to induce naïve CD4^+^ T cell proliferation (Figure 1D). Significantly, co-culture of naïve CD4^+^ T cells with CL1-5-LCAF-CM conditioned DCs led to secretion of significantly lower amounts of Th1 cytokines (IFN-γ) and increased IL-10 production when compared with CD4^+^ T cells stimulated by NHLF-conditioned DCs. In addition, the expression of Th2 cytokine IL-4, was also enhanced in CD4^+^ T cells after co-culture with CL1-5-LCAF-CM–conditioned DCs (Table 1).

**Table 1:** LCAF reduces the function of DC on Th1 priming

Cytokines	IFN-γ			IL-4			IL-5			IL-10			TNF		
**DC/T cells**	1:10	1:20	1:50	1:10	1:20	1:50	1:10	1:20	1:50	1:10	1:20	1:50	1:10	1:20	1:50
**NHLF-CM**	4009± 394.0	3289.6 ± 908.0	3223.7 ± 881.9	2.8 ± 0.5	1.1 ± 0.1	0.82 ± 0.5	7.12 ± 7.9	10.58 ± 10.6	11.538 ± 10.6	11.6 ± 9.64	15.3 ± 10.8	36.4 ± 15.7	174.69 ± 158.96	137.58 ± 72.09	83.07 ± 63.94
**CL1-5-CAF-CM**	2656.6± 365.2*	2065.1 ± 165.4*	1835 ± 645.4*	24.5 ± 11.1*	7.5 ± 1.3*	2.5 ± 0.6*	6.68 ± 2.32	10.8 ± 1.3	10.44 ± 0.3	265.6 ± 0.1*	234.6 ± 63.1*	195.8 ± 12.2*	375.36 ± 105.40	369.07 ± 71.27	257.26 ± 160.07

All results are representative of at least three independent experiments, and each value is the mean ± SD of three determinations. The results were reported as mean ± SD;

* *p* < 0.05.

### CAF increases cancer growth, migration and EMT in lung cancer

**Figure 2:**
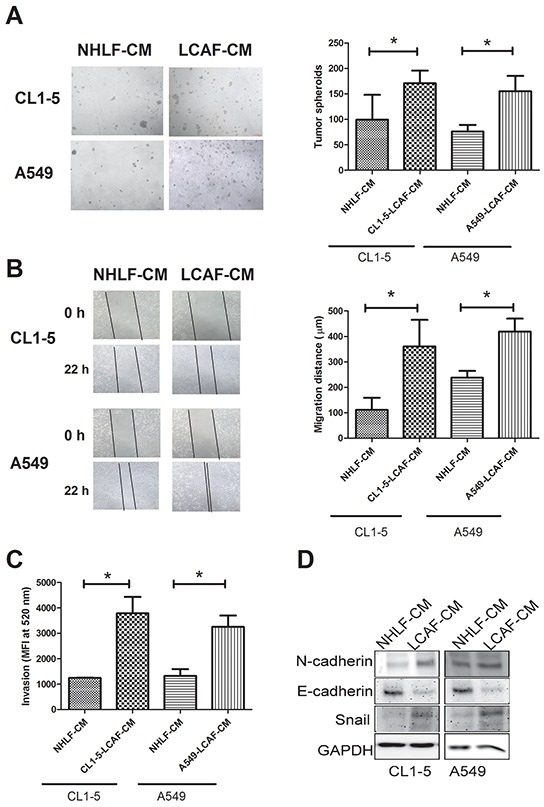
LCAF increased lung cancer proliferation, migration, invasion and EMT The CMs of LCAF increased CL1-5 and A549 spheroid formation **A.** migration **B.** and invasion **C.** The CMs of LCAF caused the EMT of lung cancer cells **D.** CL1-5 and A549 cells were cultured with CMs of CL1-5- or A549-LCAF for 21 days in an AlgiMatrix™ 3D Culture System, and the tumor spheroids counted using a microscope. CL1-5 and A549 cells were seeded into the top transwell inserts, with various CMs of LCAFs added to the bottom cells as chemo-attractants for 48 h. The invasive abilities of the CL1-5 and A549 cells were quantified by CyQUANT^®^ GR dye. CL1-5 and A549 cells were cultured with LCAF-CMs for 24 h, and the protein expression determined by Immunblot analysis. All results are representative of at least three independent experiments, with each value is the mean ± SD of three determinations. The results were reported as mean ± SD; *p < 0.05.

Growing number of studies have reported that CAF promotes cancer growth and progression [17]. We therefore assessed the effect of LCAF on lung cancer proliferation and progression. As shown in Figure 2A, compared to NHLF-CM, CL1-5- and A549-LCAF-CM increased the formation of cancer spheroids in a 3D culture system (1.72 and 2.03-fold for CL1-5- and A549-LCAF-CM, respectively). In addition, the migration and invasion of CL1-5 and A549 were also enhanced by CL1-5- and A549-LCAF-CM (3.25 and 1.76-fold for CL1-5- and A549-LCAF-CM in migration assay, 3.04 and 2.44-fold for CL1-5- and A549-LCAF-CM in invasion assay, respectively) (Figure 2B and 2C). Also, CL1-5- and A549-LCAF-CM caused epithelial-mesenchymal transition (EMT), evidence for which was supported by upregulation of mesenchymal markers (N-cadherin and snail) and downregulation of epithelial markers (E-cadherin) (Figure 2D).

### Lung cancer cells stimulate IDO1 and TDO2 expression and Kyn production in CAF

**Figure 3:**
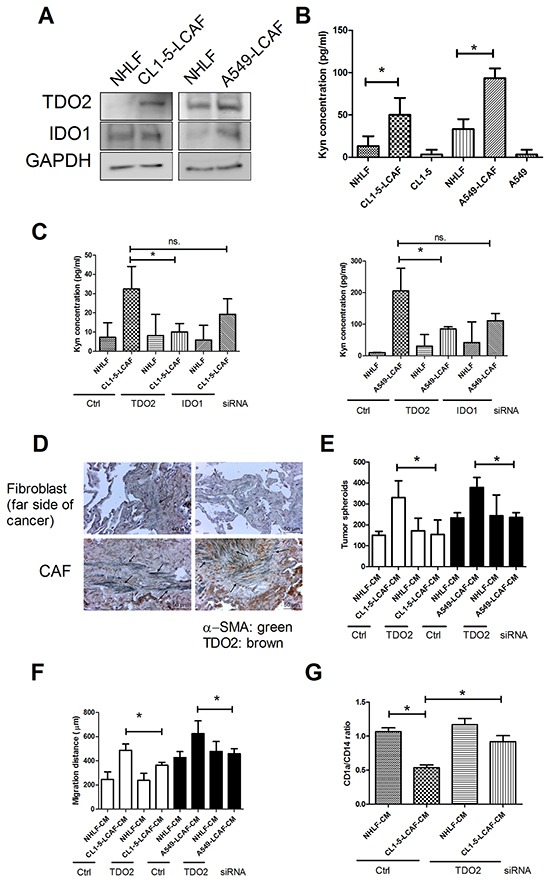
Lung cancer cells alter tryptophan metabolism in NHLF **A.** Elevated levels of TDO2 were found in LCAF. **B.** Kyn production was increased in LCAF. **C.** Knockdown of TDO2 prevented Kyn production. **D.** The expression of TDO2 in CAF of human lung cancer specimen. Inhibition of TDO2 decreased LCAF-mediated cancer spheroid formation **E.** migration **F.** and DC differentiation inhibition **G.** Wild type, control, IDO1 or TDO2 siRNA transfected-NHLF were cultured with CL1-5 cells for 24 h. The expression of various proteins was assessed by immunoblot, and the Kyn levels in supernatant of LCAF were determined by ELISA-based kits. The CMs of wild type, control or TDO2 siRNA transfected-NHLF and LCAF were collected for DC differentiation and lung cancer proliferation and migration as described in the Figure 1 Legend. The arrow indicates TDO2 and α-SMA double positive cells. All results are representative of at least three independent experiments and each value is the mean ± SD of three determinations. The results were reported as mean ± SD; *p < 0.05.

Aberrant metabolism of local stroma and the cancer cell are recognized to be critical factors contributing to cancer progression [5]. Tryptophan catabolism has been reported to suppress anticancer immunity [18]. We assessed whether lung cancer cells alter the expression of the two dioxygenase, IDO1 and TDO2, in LCAF. Figure 3A shows that increased IDO1 and TDO2 expression was found in both CL1-5- and A549-LCAF. The metabolism products Kyn were enhanced in both CL1-5 and A549-LCAF (3.75 and 2.8-fold for CL1-5- and A549-LCAF-CM, respectively) (Figure 3B). The role of IDO1 and TDO2 on Kyn production was assessed by IDO1 and TDO2 siRNA transfection. Transfection of IDO1 or TDO2 siRNA decreased the expression of IDO1 and TDO2 by 99.3% and 98.2% in fibroblast (Supplementary Figure S2A and S2B). Knockdown of IDO1 or TDO2 by specific siRNA transfect shows that TDO2 inhibition prevents the production of Kyn in LCAF. In contrast, blockade of IDO1 by siRNA only slightly affects the production of Kyn in LCAF (Figure 3C). Furthermore, upregulation of TDO2 was found in LCAF adjacent to the cancer region, when compared to the fibroblast on the far side of cancer, in specimens taken from patients with lung cancer (Figure 3D).

To further investigate the role of Kyn on immune suppression and cancer progression, we assessed the effect of Kyn on DCs differentiation and cancer proliferation as well as migration. Kyn inhibited DCs differentiation from CD14+ monocytes (Supplementary Figure S3A). Kyn-conditioned DCs showed impaired ability to induce naïve CD4^+^ T cell proliferation (Supplementary Figure S3B). Significantly, co-culture of naïve CD4^+^ T cells with Kyn- conditioned DCs led to secretion of significantly lower level of IFN-γ and, increased IL-4 and -10 production when compared with CD4^+^ T cells stimulated by DCs (Supplementary Figure S3C to S3E). In addition, Kyn also increased cancer spheroids formation, migration and EMT (Supplementary Figure S4A to S4C) in both CL1-5 and A549 cells, suggesting that Kyn promotes cancer growth and progression. Moreover, knockdown of fibroblasts' TDO2 by siRNA also prevents the impairment of CL1-5-LCAF on DC differentiation and Th2 response trigger, together with cancer migration and proliferation (Figure 3E to 3G, Supplementary Figure S5A to S5C).

**Figure 4:**
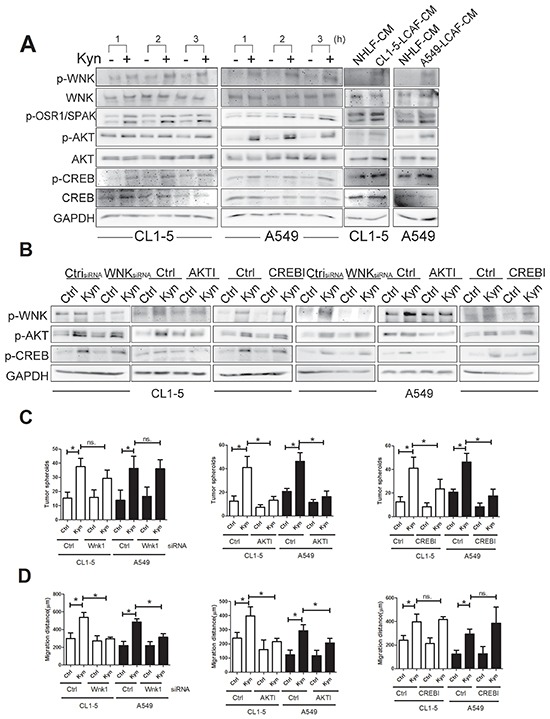
AKT/CREB and AKT/WNK1 pathways mediated Kyn-mediated cell proliferation and migration **A.** LACF-CM and Kyn activate AKT, CREB and WNK1. **B.** AKT is the upstream regulator of CREB and WNK. **C.** CREB is involved in Kyn-mediated cell proliferation. **D.** WNK is the major factor to increase cell migration induced by Kyn. CL1-5 and A549 cells were pretreated, with or without PI3K inhibitor (LY294004) or CREB inhibitor for 1 h, and LCAF-CMs (20%) and Kyn (50 mM) were added for another 3h. The knockdown of WNK was carried out by WNK1 shRNA transfection. Control or WNK1 shRNA transfected CL1-5 and A549 cells were committed to migration analysis, as described in Figure 1. Protein expression was assessed by immunoblot analysis. All results are representative of at least three independent experiments and each value is the mean ± SD of three determinations. The results were reported as mean ± SD; *p < 0.05.

Next, we further investigated the molecular mechanism of Kyn on the enhancement of lung cancer progression. We created the phosphorylated protein profile of Kyn-treated CL1-5 cells by phosphokinase array. The data shows that Kyn increased the phosphorylation of AKT (S473), With-No-K (Lysine) kinase 1 (WNK1) (T60), and cAMP response element-bindingprotein (CREB) (S133) (Supplementary Figure S6). Immunoblot also showed that LCAF-CMs and Kyn increased the activation of AKT, CREB, WNK and WNKs' targets oxidative stress responsive 1 (OSR1) (T325), Ste20 Protein Kinase (SPAK) (T373) in CL1-5 and A549 cells (Figure 4A). To investigate the order of sequential events, AKT and CREB inhibitors and WNK shRNA were used to assess the phosphorylated status of AKT, CREB and WNK. Transfection of WNK shRNA decreased the expression of WNK by 57.8% and 67.8% in CL1-5 and A549 cells, respectively (Supplementary Figure S7). AKT inhibitor decreased the phosphorylation of CREB and WNK, whereas the CREB inhibitor and WNK knockdown did not affect the phosphorylation of AKT (Figure 4B), showing that AKT is the upstream regulator of CREB and WNK. Moreover, CREB inhibitor markedly inhibited Kyn-driven tumor spheroid formation, but did not decreased Kyn-induced cell migration. In contrast, WNK siRNA decreased the Kyn-mediated cell migration, but WNK siRNA did not inhibited tumor spheroid formation. As expected, AKT inhibitor prevented Kyn-mediated tumor spheroid formation and migration (Figure 4C and 4D). These findings indicate that AKT/CREB and AKT/WNK signaling pathways play a role in the regulation of tumor spheroid formation and cell migration, respectively.

### Galectin-1 is the major factor contributing to the upregulation of TDO2 in LCAF

**Figure 5:**
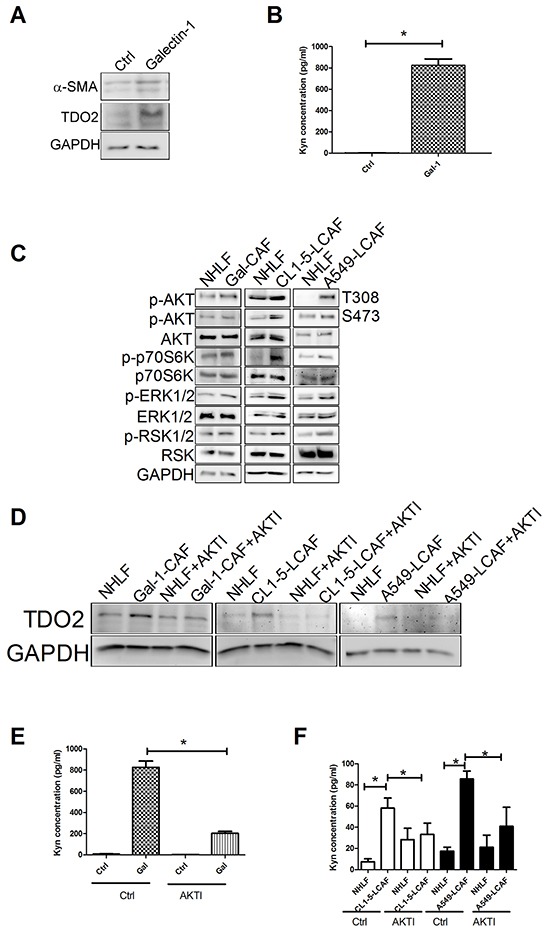
Galectin-1 increased the activation of LCAF by AKT pathway **A.** rhgalectin-1 increased TDO2 and α-SMA expression (A) and Kyn production **B.** in NHLF. **C.** rhgalectin-1 increased the activation of AKT and ERK signaling pathways. AKT inhibitor decreased rhgalectin-1 induced TDO2 upregulation **D.** and Kyn production **E.** in NHLF. **F.** AKT inhibitor reduced Kyn production in CL1-5- and A549-LCAF. NHLF cells were treated with rhgalectin-1 (1 μg/ml) for 3 h (for AKT and ERK) or 24 h (for TDO2 and α-SMA). The expression of various protein was assessed by immunoblot. The level of Kyn in culture media was determined by ELISA. For blocking experiment, NHLF cells were pretreated with LY294002 (10 μM) or MEK (PD98059, 10 μM) inhibitors for 1 h. All results are representative of at least three independent experiments and each value is the mean ± SD of three determinations. The results were reported as mean ± SD; *p < 0.05.

Our previous studies reported that cancer-derived galectin-1 contributes to DC impairment [14], and that elevated levels of IDO, an important immunosuppression enzyme, are expressed in tolerogenic DCs in the cancer microenvironment [19]. Therefore, we assessed whether galectin-1 regulates the expression of TDO2. As shown in Figure 5, addition of recombinant human (rh) galectin-1 increases the expression of α-SMA and TDO2, and further enhances the production of Kyn by a 670.2-fold in NHLF (Figure 5A and 5B). Knockdown of galectin-1 loses the inductive effect of CL1-5 cancer cells on TDO2 expression and Kyn production in LCAF (Supplementary Figure S8A and S8B), suggesting that lung cancer-derived galectin-1 is responsible for TDO2 induction in LCAF.

To investigate the molecular mechanism of galectin-1 on TDO2 induction, the phosphorylated protein profile of galectin-1-treated NHLF cells was assessed by phosphokinase array. Compared to NHLF, galectin-1 treatment enhanced the phosphorylation of AKT, p70S6K, ERK1/2/, RSK1/2, STAT1 and Pyk2. Immunoblot data shows that galectin-1 increases the activation of AKT (AKT, p70S6K) and ERK1/2 (ERK1/2 and RSK1/2) signaling pathways (Supplementary Figure S9A and Figure 5C). Also, co-culture of NHLF with CL1-5 and A549 cells increases the activation of AKT and ERK1/2 signaling (Figure 5C). Inhibition of AKT by specific inhibitor prevents the galectin-1-mediated TDO2 upregulation and Kyn production in NHLF (Figure 5D and 5E), but not ERK inhibitor (Supplementary Figure S9B and S9C). Similar results are also found in NHLF co-cultured with CL1-5 and A549 cells (Figure 5D and 5F), suggesting that AKT signaling is associated with TDO2 upregulation in LCAF.

### Targeting TDO reverses immune response and decreases lung cancer metastasis *in vivo*

**Figure 6:**
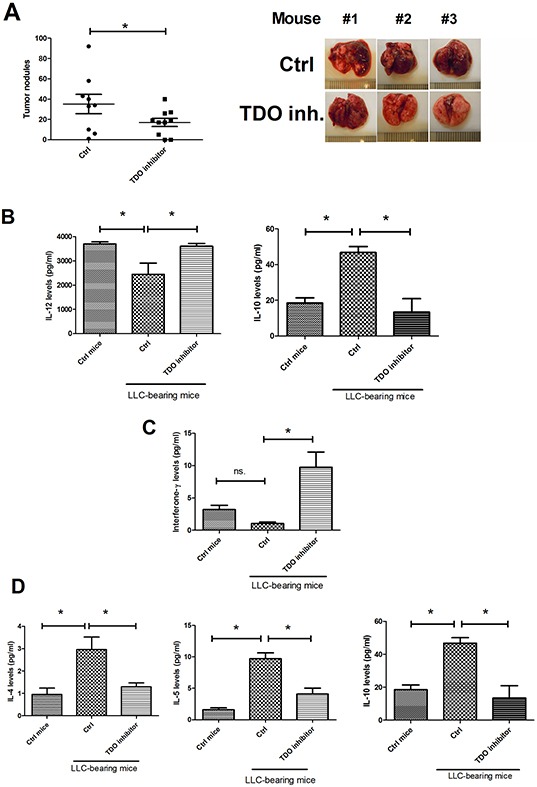
TDO inhibitor decreased cancer metastasis and improved anticancer-immunity in vivo **A.** TDO inhibitor decreased lung metastasis. TDO inhibitor increased IL-12 and decreased IL-10 expression in cancer-infiltration DCs **B.** TDO inhibitor enhanced Th1 **C.** and decreased Th2 cytokines' expression in CD4 T cells **D.** LLC cells were implanted into C57BL mice via tail vein injection. The mice were then randomly divided into two groups: the TDO2 inhibitor 680c91 -treated group was given ip daily with 680c91 (8 mg/kg per day), while the control group was given an equal volume of normal saline. Tumor-bearing mice were sacrificed 18 days after transplantation. The mice's lungs were removed and the metastatic tumor nodules counted. DC (CD11c+, F4/80-) and CD4+ T cells were isolated from the lungs. DCs and T cells were cultured in RPMI 1640 for 1 day, and cytokine levels were assessed by MILLIPLEX kits. Each value is the mean ± SD of three determinations. *Significant difference between the two test groups, as analyzed by Student's *t* test (*p*<0.05).

This study assessed whether targeting TDO2 could decrease lung cancer development. Consequently, we treated mice with TDO inhibitor (8 mg/kg) and assessed the status of lung cancer metastasis in mice. After 16 days of treatment, the mean number of tumor nodules was 31.28 in the control group, whereas TDO inhibitor treatment proved significantly effective in decreasing the occurrence of tumor nodules (14.0) (Figure 6A). Administration of TDO inhibitor improves the immune tolerance of DC, as it supported by increasing IL-12 and decreasing IL-10 expression in DCs (CD11C^+^F^−^/40^−^) isolated from the lungs of LLC-bearing mice (Figure 6B). In addition, TDO inhibitor also increase Th1 cytokines (IFN-γ) and reduces Th2 cytokines (IL-4, IL-5 and IL-10) in CD4^+^ T cells isolated from the lungs of LLC-bearing mice (Figure 6C and 6D).

## DISCUSSION

CAFs have been indicated to be is necessary for the entire process of cancer development, from the pre-neoplastic state until the terminal stage of cancer metastasis [20]. Interaction within the TME is not restricted to paracrine signaling between cancer cells and CAFs, but also takes place between different residents, including immune cells. The present study shows that Trp metabolite Kyn, produced by LCAF, exerts an immune inhibitory effect and a pro-tumorigenetic effect on the various stages of cancer development, including cell proliferation, migration and EMT. The crosstalk of cancer cells, CAFs and immune cells in TME studied here confirms that multiple targets may be a promising strategy for increasing the efficiency of immunotherapy or target therapy.

Our study reveals that LCAF contributes to cancer immunity evasion in lung cancer. DCs generated in the presence of LCAF-CM failed to downregulate the monocytic marker CD14 and upregulate DC marker CD1a and IL-12 expression. The dysfunction of LCAF- and Kyn derived DCs was revealed by reflecting the failure to elicit effective T cell activation, and reprogram T response toward the T helper 2 (Th2)-cell-like phenotype. Knockdown of TDO2 reverses the immunosuppressive effect of LCAF, indicating that Kyn is responsible for LCAF-mediated immune inhibition. Moreover, TDO2 inhibitor improves DCs' differentiation and function, and restores T cell response toward Th1 phenotype in mice. Kyn has been indicated to be an immunosuppressive regulator of innate and adaptive immunity, and to contribute to immune tolerance during cancer development. Kyn reprograms the differentiation of naïve CD4+ T-helper cells favoring a regulatory T cells phenotype [21]. This study is the first to demonstrate an unexplored but important impact of cancer-associated fibroblasts on the development of immunosuppressive TME in lung cancer.

Emerging evidence implicates a promotion of Kyn on cancer development and patient prognosis [10, 22]. Kyn has been indicated to be responsible for tumor spheroids and the motility of malignant glioma cells by activating aryl hydrocarbon receptor (AhR) receptor and related signaling pathwasy [21]. Overexpression of AhR accelerates the proliferation of lung cancer A549 cells [22]. In addition, Kyn has been indicated to increase E-cadherin degradation, which is a critical process inbecoming more migratory [6]. In this study, we provide evidence that LCAF contributes to the development of tumor cells via the Kyn paracrine effect. This is supported by the finding that knockdown of TDO2 in LCAF prevents the stimulation of LCAF in promoting cancer proliferation and movement.

The pivotal role of CREB signaling on cell survival and proliferation in human cancers has long been established [23]. AKT/PKB potently induces S133 phosphorylation of CREB and promotes recruitment of CBP, and increases transcription of cell division target genes, such as cyclins and other oncogenic transcription factors [24–26]. Here, we show thatKyn increases the activation of AKT, which in turn increases phosphorylation of CREB, resulting in the enhancement of cancer proliferation. Inhibition of AKT by specific inhibitor prevents Kyn-mediated CREB phosphorylation, and blockade of CREB decreases Kyn-mediated cell proliferation, revealing that the AKT/CREB axis contributes to cancer growth by the deteriorating cycle of lung cancer-fibroblast.

WNK1 is a serine/threonine kinase which plays an initial role in cancer development, including tumorigenesis, cell proliferation, migration, invasion and metastasis [27, 28]. WNK1 has been indicated to regulate endothelial cell migration, invasion and angiogenesis [28]. AKT can phosphorylate WNK1 at threonine residue 60, and facilitate WNK1 to form a complex with serine/threonine-protein kinase [29–31]. Activated WNK1 phosphorylates its target OSR1 at S325 residue, which in turn regulates the EMT modulator Slug expression [28, 29]. In this study, we found that LCAF-CM and Kyn increased the phosphorylation of WNK1 activation in an AKT dependent manner. Enhanced WNK1 activity was also supported by two WNK1 targets SAPK and OSR1 phosphorylation at T373 and T325, respectively. Moreover, knockdown of WNK1 by siRNA transfection prevented cancer migration mediated by LCAF-CM and Kyn. These findings suggest that LCAF-derived Kyn activates AKT, resulting in the triggering of WNK1-mediated cancer progression in lung cancer.

Galectin-1 has already been implicated in the modulation of several processes required for cancer development, such as cancer stem cell increase and cancer metastasis [32–34]. Galectin-1 also promotes cancer evasion of the host's immune response by causing DC anergy and T cell apoptosis [34, 35]. Elevated Galectin-1 has been found in CAF and contributes to CAF activation and CAF-mediated cancer progression [15, 36]. By expanding upon previous studies, results of the present study show that galectin-1 is involved in lung cancer-mediated changes in switching switch of fibroblasts to myofibroblasts, and Trp metabolism. Knockdown of galectin-1 in cancer cells decreased the upregulation of TDO2 and consequently reduced Kyn production in LCAF. Furthermore, the upregulation of TDO2 in activated fibroblasts induced by lung cancer-derived galectin is in an AKT-dependent manner, which enhances IDO expression [37, 38]. Contributions from other soluble factors in TEM cannot be excluded, but our series of studies have observed that galectin-1 secreted by cancer is a primary factor responsible for immune surveillance, by both direct and indirect influence, mediated by cancer-galectin-1-CAF axis on DCs dysfunction.

**Figure 7:**
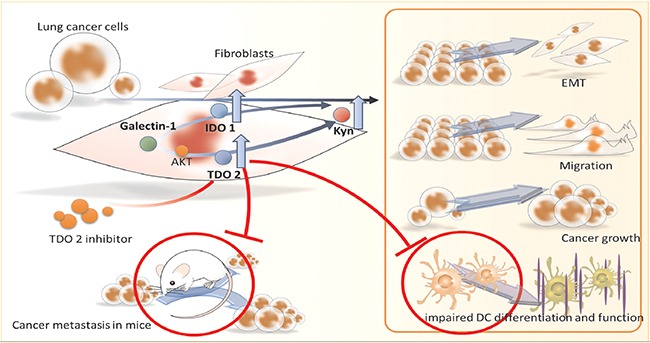
Scheme of proposed lung cancer-derived galectin-1 contributes to cancer associated fibroblast-mediated cancer progression and immune inhibition by TDO2/kynurenine axis

Taken together, the findings here suggest interaction between lung cancer and fibroblast-derived abnormal tryptophan metabolism, which promotes lung cancer progression. Kyn, produced by cancer associated fibroblasts, exacerbates cancer growth, migration, and metastasis (Figure 7). Targeting TDO2 significantly decreases the incidence of cancer progression and restores anti-cancer immunity in mice. The collaborative interactions of cancer and their supportive stroma fibroblasts provide valuable information regarding the dynamics and reciprocal communication and pathologic impacts of abnormal tryptophan metabolism. Selective inhibition on multiple targets of stroma may be a novel modality for lung cancer treatment.

## MATERIALS AND METHODS

### Lung cancer cells and co-culture

Primary normal human lung fibroblasts (NHLF) were obtained from Lonza (Walkersville, MD) and cultured in fibroblast basal medium (FBM) supplemented with hFGF-B, insulin, gentamicin, amphotericin B, and 2% fetal bovine serum (FBS) (Lonza). All experiments were conducted from passage 2-6. CL1-5 human lung adenocarcinoma cell lines were generously provided by Dr. Pan-Chyr Yang (Department of Internal Medicine, National Taiwan University Hospital), and cultured in RPMI 1640 supplemented with 10% FBS and 1% penicillin-streptomycin (Lonza). Human lung cancer cells A549 were obtained from the American Type Culture Collection (number CCL-185) and were cultured in F-12K culture medium (Invitrogen Co., Carlsbad, CA) supplemented with 10% FBS and 1% penicillin-streptomycin (Lonza). To obtain CL1-5 and A549-conditioned media (CM), the cells were seeded as 2 × 10^6^ cells/100 mm dish and cultivated for 24 hours. The medium was replaced and the supernatants (conditioned media; CM) harvested after 48 h of incubation. Kyn were obtained from Sigma–Aldrich (St Louis, MO). AKT and CREB inhibitors were obtained from (EMD Millipore, Billerica, MA).

For the co-culture of NHLF and lung cancer cells, 1.5×10^5^ NHLF and lung cancer cells (CL1-5 or A549 cells) were seeded in transwell inserts (pore size: 3μm) and a 6 well plate, then cultured for 24 or 48 h. The medium was collected and defined as NHLF-CM, CL1-5-LCAF-CM and A549-LCAF-CM). All CMs were frozen and stored at -80°C, and thawed singly for the study.

### Generation of monocyte-derived DCs and allogeneic MLR

CD14^+^ monocytes and naïve CD4^+^ T cells were purified from peripheral blood mononuclear cells (PBMCs) obtained from healthy consenting donors, as reported in the previous study.^12^ DCs were generated by culturing CD14^+^ monocytes in RPMI 1640 medium containing 10% FBS (Invitrogen, Carlsbad, CA), 20 ng/mL GM-CSF, and 10 ng/mL IL-4 (R&D Systems, Minneapolis, MN) for 5 days. The medium was replaced with fresh medium containing GM-CSF and IL-4 on day 3. For maturation of DCs, immature DCs were stimulated with LPS (100 ng/ml) after priming with interferon-γ (IFN-γ) for 3 h. CL1-5 and A549-LCAF conditioned DCs were generated by culturing CD14^+^ monocytes in RPMI 1640 medium containing FBS, GM-CSF, and IL-4 presenting in CL1-5-LCAF-CM or A549-LCAF-CM (20%), then stimulated as described above.

MLR was carried out by culturing naïve CD4^+^ T cells for a set number of mature DCs (10^4^cells/well) in 96-well plates for 5 days. Human T cells were purified from PBMCs obtained from healthy consenting donors using immunomagnetic CD4 naive T cell MACS beads and a MACS LS column (Miltenyi Biotec Inc, Auburn, CA). The T cell proliferation was assessed by BrdU Cell Proliferation Kit (EMD Millipore, Billerica, MA), as described in a previous study [14].

### Measurement of secreted factors and Kyn

Supernatants from DCs and T cells were collected. Various cytokines were determined by MILLIPLEX kits (EMD Millipore). Levels of Kyn in the supernatant LCAFs were determined by Human Kynurenine (KYN) ELISA Kit (Cusabio Biotech Co., Ltd., Wuhan, China).

### Flow cytometry analysis

The DCs derived from CD14^+^ monocytes were stained by antibodies against human CD14 labeled by FITC and CD1a labeled by phycoerythrin (PE) (BD Biosciences, San Jose, CA). The expression of each molecule was analyzed using an Acuri C6 flow cytometer (BD Biosciences).

### Analysis of cell proliferation, migration and invasion

Cell proliferation was assessed by AlgiMatrix 24-well plates (Invitrogen, San Diego, CA) according to the manufacturer's instructions. The migration of CL1-5 and A549 cells was assessed by scratch wound-healing assay. Cells were allowed to grow to full confluence in 24-well plates. The following day, a uniform scratch was made down the center of the well using a micropipette tip, followed by washing once with PBS. Various CMs and Kyn were added to the respective wells for the indicated times. Photographic imaging was performed using a Nikon inverted microscope (Nikon, Melville, NY). Cell invasion assays were conducted using the QCM™ 24-well Cell Invasion System(EMD Millipore). NHLF, CL1-5-LCAF-CM and A549-LCAF-CM were added to the bottom wells for 24 h as chemo-attractant. Fluorescence of the invading cells was read using a fluorescence plate reader at excitation/emission wavelengths of 485/530 nm.

### Immunoblot/phospho-kinase-antibody array/immunohistochemical reactions (IHC)

Cells were lysed on ice for 15 min by RIPA lysis reagent (EMD Millipore). The cell lysate was centrifuged at 14,000 × *g* for 15 min and the supernatant fraction harvested for immunoblot. Equivalent amounts of protein were resolved by SDS-PAGE (8-12%) and transferred to PVDF membranes (EMD Millipore). After blocking for 2 h in 5% non-fat dry milk in Tris-buffered saline, the membrane was incubated overnight with the desired primary antibody. The membrane was then treated with peroxidase-conjugated secondary antibody, and the levels of various proteins detected using an enhanced chemiluminescence kit (EMD Millipore). Antibodies to AKT, phspho-AKT, ERK1/2, phospho-ERK1/2, p70S6K, phosphor-p70S6K, WNK, phospho-WNK, CREB, phosphor-CREB, Snail and GAPDH were from Cell Signaling (Beverly, MA, USA). Antibodies to N-cadherin and E-cadherin were from BD Biosciences. Antibodies against TDO2 and IDO1 were from Abcam (Cambridge, UK). Antibodies against phosphor-OSR1/SPAK from EMD Millipore. Antibodies against α-smooth muscle actin (α-SMA) were from Sigma–Aldrich (St Louis, MO). The phosphorylation profile of 43 kinases was assessed by Phospho-Kinase Array Kit (R&D Systems).

Non-cancerous and cancerous lung tissue specimens obtained from human lung cancer patients were embedded in Non-cancerous and cancerous lung tissue specimens obtained from human lung cancer patients. All IHCwere performed on 5-μm-thick paraffin sections. The expressions of a-SMA and TDO2 antigen were demonstrated using mouse monoclonal anti-a-SMA (dilution 1:25, Abcam Ltd. Cambridge, UK) and anti-TDO2 (dilution 1:100, Biobyt Ltd. Cambridge, UK) antibodies, respectively. All of the sections were counterstained with hematoxylin.

### Gene knockdown of TDO2 and IDO1 by siRNA transfection

Knockdown of TDO2 or IDO1 in NHLF cells was performed using an ON-TARGET smart pool control, IDO1 and TDO2 siRNA (Thermo Fisher Scientific, Waltham, USA). Efficacy of the IDO1 and TDO2 siRNA transfection was assessed by qRT-PCR. Knockdown of WNK1 in CL1-5 and A549 cells was performed using a lentiviral expression system provided by the National RNAi Core Facility (Taipei, Taiwan).

### Animal models

Lewis lung carcinoma (LLC, 1×10^6^/mice) cells were transplanted via tail vein into C57BL/6 mice. TDO inhibitor 680c91 (8 mg/kg per day intraperitoneal injection.)(Sigma–Aldrich (St Louis, MO). or an equal volume of vehicle (0.2% DMSO plus 40% PEG 400 in normal saline) was administered. The animals were sacrificed on day 18 after LLC transplantation, and the number of tumor nodules was recorded for the analysis of lung cancer incidence. The digested tissues were filtered through a 70-μm cell strainer and washed with RPMI 1640 medium. CD11c^+^ DCs were isolated from the cell suspension by CD11c magnetic beads (Miltenyi Biote).

### Immunohistochemical reactions (IHC)

Non-cancerous and cancerous lung tissue specimens obtained from human lung cancer patients were embedded in Non-cancerous and cancerous lung tissue specimens obtained from human lung cancer patients. All IHCwere performed on 5-μm-thick paraffin sections. In brief, the sections were deparaffinized in xylene and rehydrated, and then incubated in target retrieval solution (DAKO, Carpinteria, CA) at an autoclave for 8 min in order to retrieve the antigens. The activity of endogenous peroxidase was blocked by 10 minutes incubation with 3% solution of H_2_O_2_. The expressions of α-SMA and TDO2 antigen were demonstrated using mouse monoclonal anti-a-SMA (dilution 1:25, Abcam Ltd. Cambridge, UK) and anti-TDO2 (dilution 1:100, Biobyt Ltd. Cambridge, UK) antibodies, respectively. The sections were incubated with the primary antibodies overnight at 4°C. The studied antigens were then visualized using biotinylated antibodies and streptavidin, conjugated with horseradish peroxidase. Diaminobenzidine Dako Cytomation (Glostrup, Denmark)) served as the substrate, and all of the sections were counterstained with hematoxylin.

### Statistical analysis

Data were expressed as means ± SD. Statistical analyses between control and experimental groups were analyzed by an unpaired Student's *t*-test. Multiple comparisons were evaluated by one-way ANOVA, and differences in the mean values among groups were determined by a Turkey *post hoc* analysis. *p* values < 0.05 were considered to be statistically significant.

## SUPPLEMENTARY FIGURES


