# Supplementary material for: Lung cancer-derived galectin-1 contributes to cancer associated fibroblast-mediated cancer progression and immune suppression through TDO2/kynurenine axis
Source: Oncotarget. 2016 Mar 30;7(19):27584–98. doi: 10.18632/oncotarget.8488 (PMC5053673; doi:10.18632/oncotarget.8488)
Supplement: Supplementary file 1 [file oncotarget-07-27584-s001.pdf]

SUPPLEMENTARY FIGURES

A

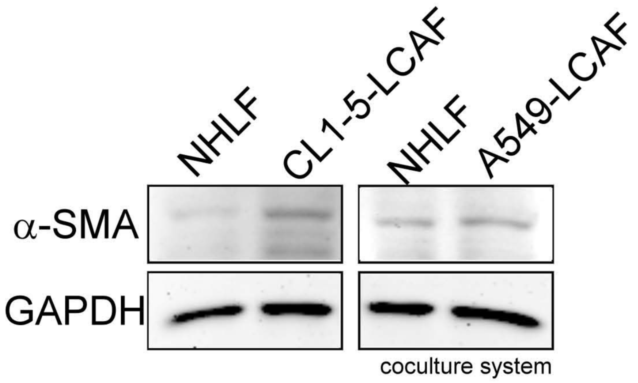

B

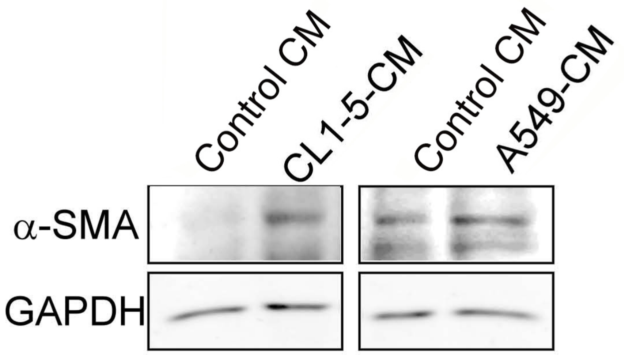

Supplementary Figure S1: Lung cancer increases the activation of normal primary lung fibroblasts (NHLF). A. co-culture of lung cancer cells with NHLF. **B.** The CMs of lung cancer cells increased the expression of a-SMA in NHLF. NHLF cells were seeded into a 6 well plate and lung cancer cells (CL1-5 and A549) were seeded in a transwell insert. The co-culture system was maintained for 24 h. Alternatively, NHLF were treated with or without CL1-5- or A549-CM (20%) for 24 h. Protein expression was assessed by Immunoblot analysis. All results are representative of at least three independent experiments.

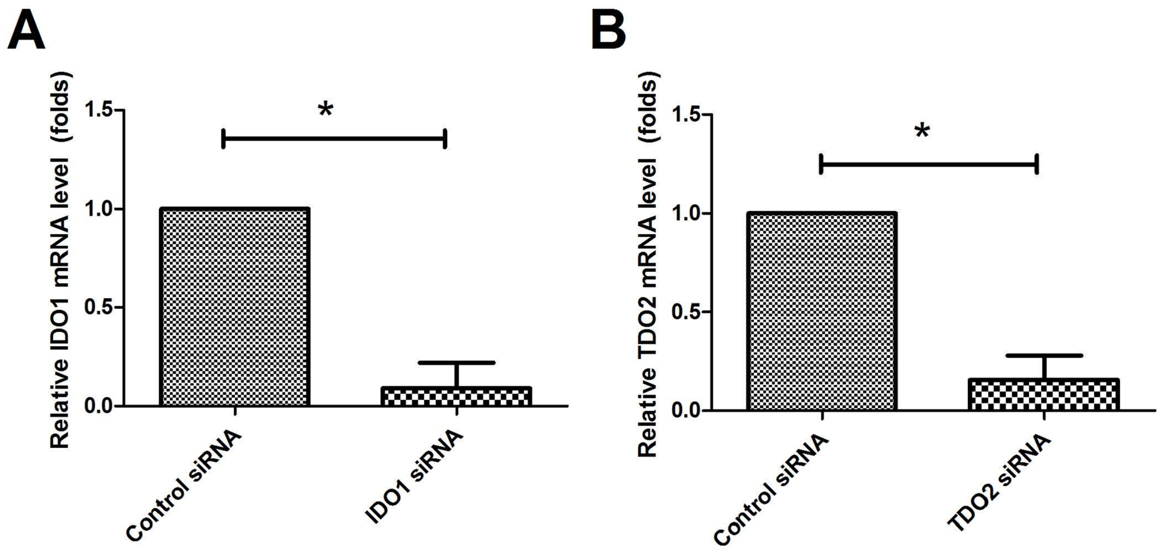

Supplementary Figure S2: The knockdown efficacy of IDO1 and TDO2 siRNA. A. IDO siRNA transfection decreased the expression of IDO1. **B.** TDO2 siRNA transfection decreased the expression of TDO2. Cell were transfected with ON-TARGET smart pool control, IDO1 and TDO2 siRNA (20 nM). The expression of f IDO1 and TDO2 was assessed by qRT-PCR.All results are representative of at least three independent experiments and each value is the mean ± SD of three determinations. The results were reported as mean ± SD; $*p < 0.05$.

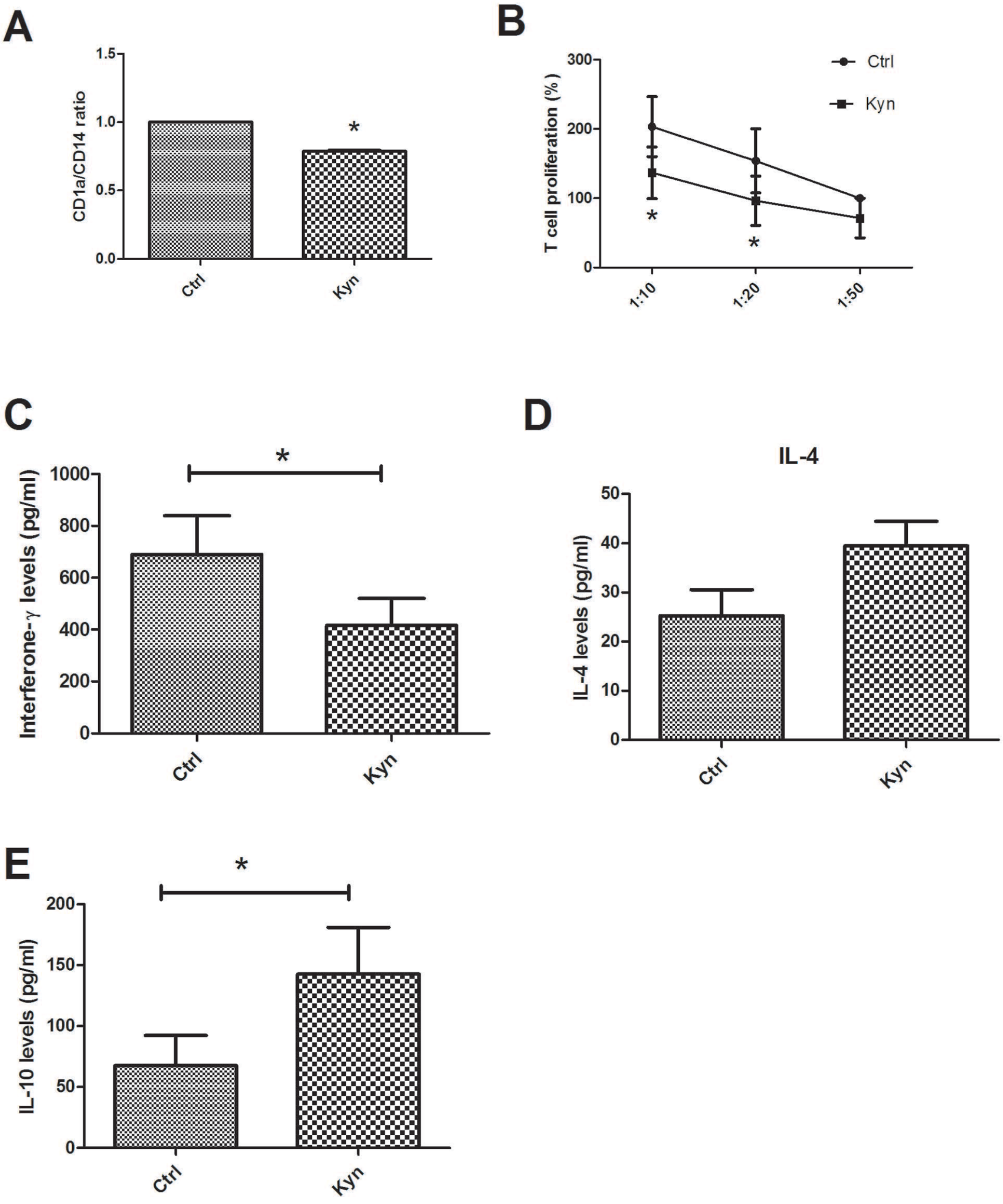

Supplementary Figure S3: Kyn impaired DC differentiation and function. A. Kyn decreased the ratio of CD1a/CD14 in DCs. **B.** Kyn-derived DCs exhibited lower T cell proliferation. The IFN-γ **C.** IL-4 **D.** and IL-10 **E.** expression of CD4 T cells. $CD14^{+}$ monocytes were cultured with Kyn (100 µM), GM-CSF (20 ng/ml) and IL-4 (20 ng/ml) for 5 days. After stimulation by IFN and LPS, mature DCs were co-cultured with naïve CD4 T cells for another 5 days. The T cell proliferation were assessed by BrdU cell proliferation kit, and the levels of cytokines in the DC and T cell supernatants were determined by MILLIPLEX MAP kits. All results are representative of at least three independent experiments and each value is the mean ± SD of three determinations. The results were reported as mean ± SD; $*p < 0.05$.

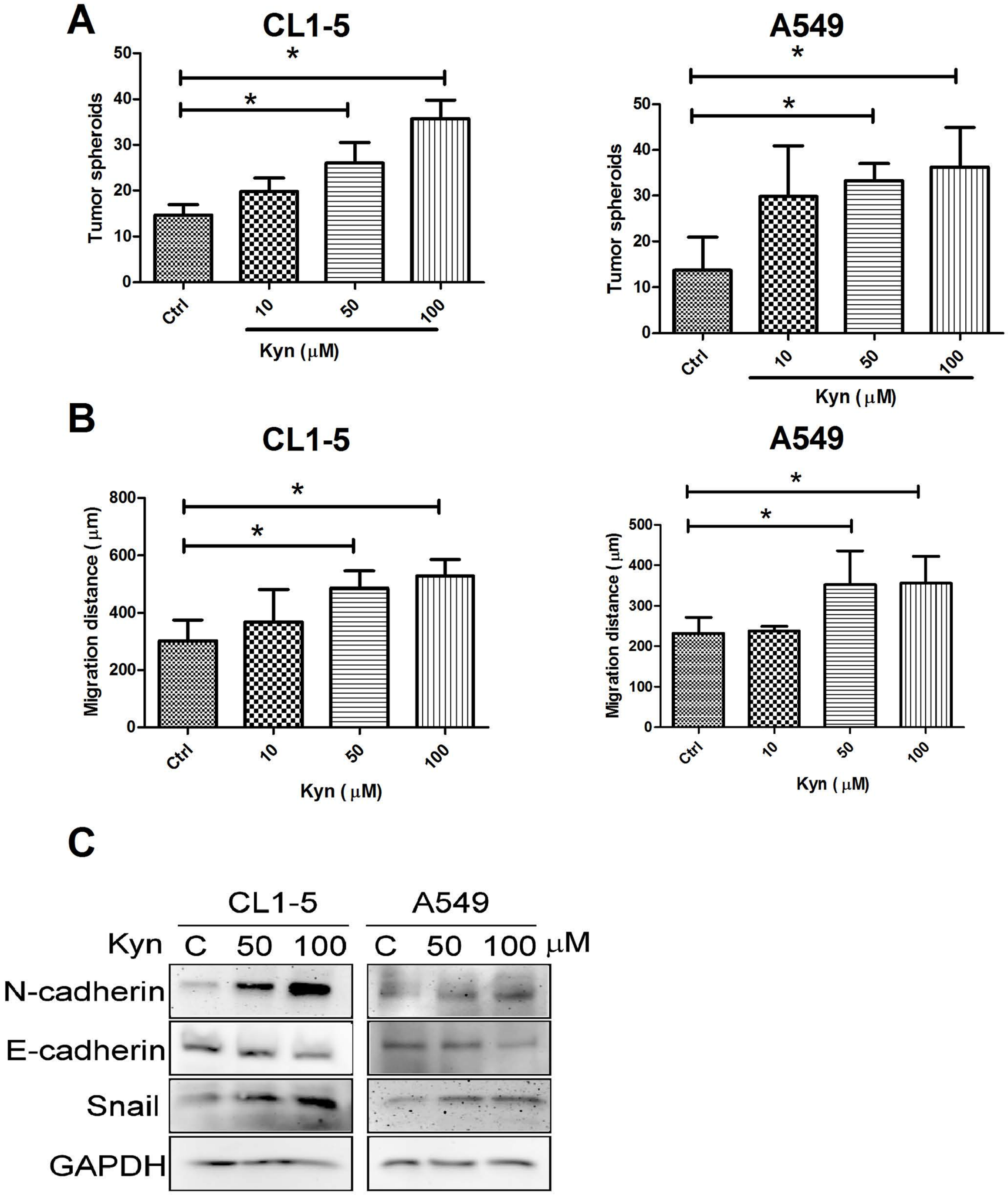

Supplementary Figure S4: Kyn increased lung cancer proliferation and progression. Kyn enhanced tumor spheroid formation **A.** migration **B.** and decreased E-cadherin expression **C.** in both CL1-5 and A549. Lung cancer cells were cultured with Kyn (10, 50 or 100 μM). Protein expression was assessed by Immunoblot. The migration of cells was assessed by scratch wound-healing assay. Proliferation of CL1-5 and A549 cells was assessed using an AlgiMatrix™ 3D culture system. All results are representative of at least three independent experiments and each value is the mean ± SD of three determinations. The results were reported as mean ± SD; *$p < 0.05$.

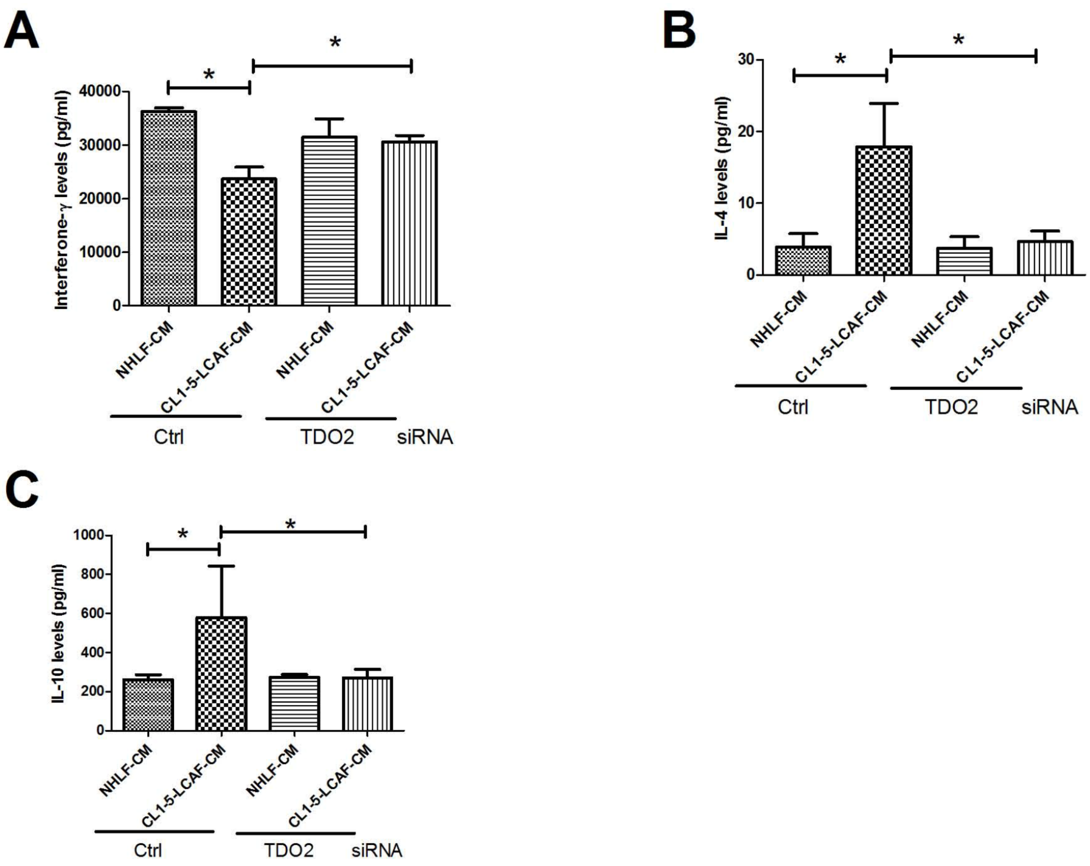

Supplementary Figure S5: The role of TDO2 on LCAF-mediated Th2 response. Inhibition of TDO2 restored IFN-γ **A.** and decreased IL-4 **B.** and IL-10 **C.** expression in LCAF-associated DC conditioned T cells. MLR was carried out by culturing naïve $CD4^+$ T cells for a set number of mature DCs (10^4 cells/well) in 96-well plates for 5 days. The levels of cytokines in the T cell supernatants were determined by MILLIPLEX MAP kits. All results are representative of at least three independent experiments and each value is the mean ± SD of three determinations. The results were reported as mean ± SD; *$p < 0.05$.

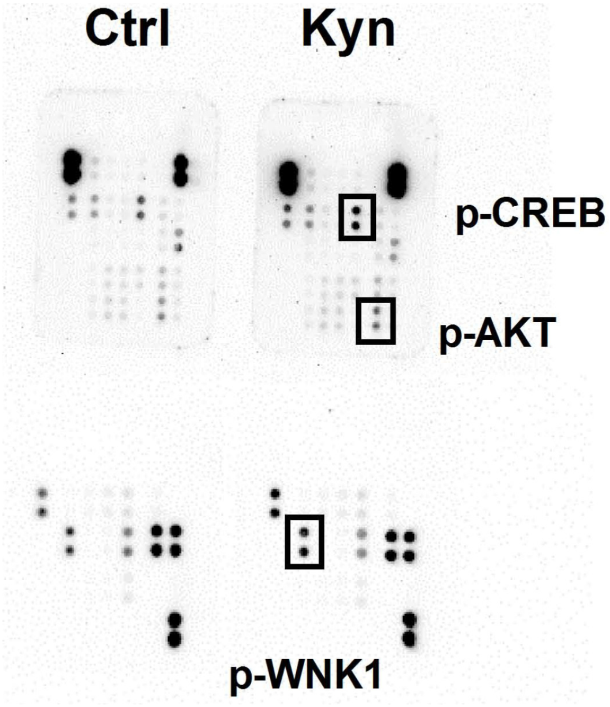

Supplementary Figure S6: The phosphorylated kinase profile of Kyn-treated CL1-5 cell. CL1-5 cells were treated with Kyn (100 μM) for 3 h. The expressions of various kinases were assessed by Human Phospho-Kinase Array.

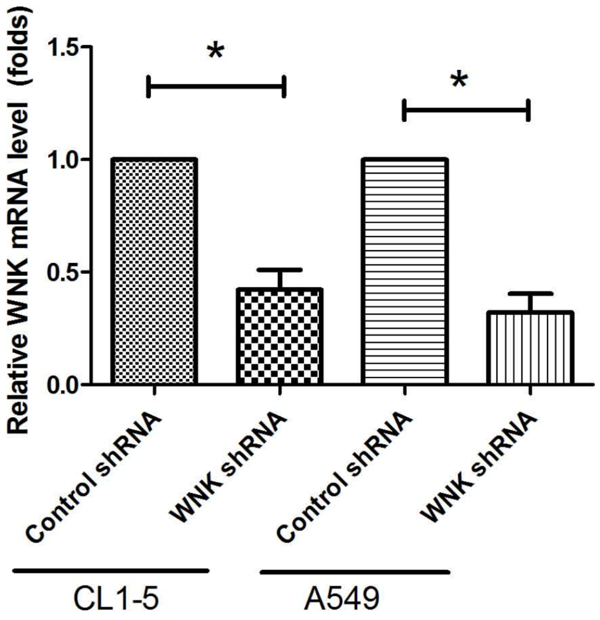

Supplementary Figure S7: The knockdown efficacy of WNK shRNA. Cell were transfected either with control plasmid or WNK shRNA. The expression of WNK1 was assessed by qRT-PCR. All results are representative of at least three independent experiments and each value is the mean ± SD of three determinations. The results were reported as mean ± SD; *$p < 0.05$.

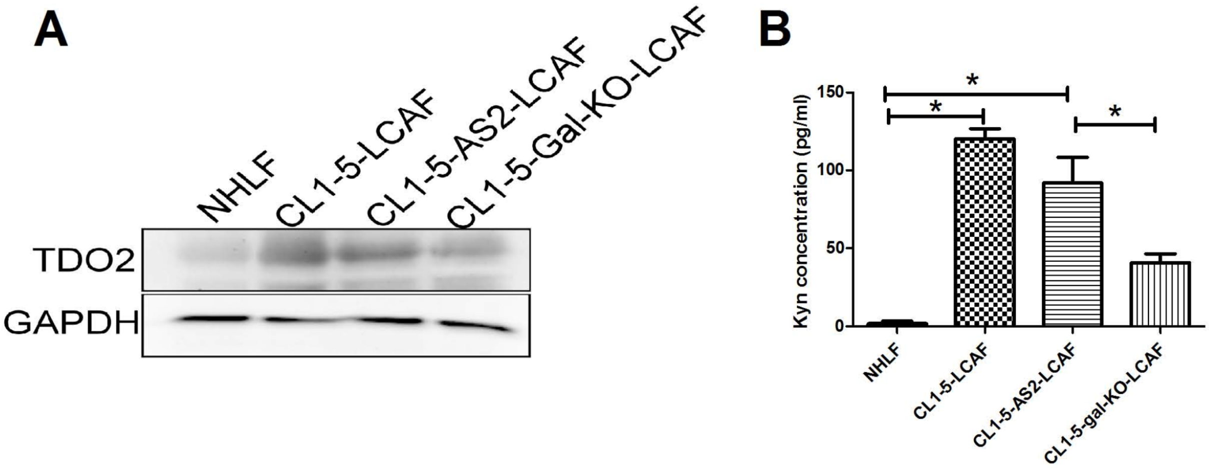

Supplementary Figure S8: Galectin-1 is the mediator of CAF activation. Knockdown of galectin-1 decreased the TDO2 upregulation **A.** and Kyn production **B.** in CL1-5-stimulation CAF. NHLF cells were treated with CMs of control or galectin-1 shRNA-transfected CL1-5 (20%) for 24 h, the expression of TDO2 and Kyn was assessed by Immunoblot and ELISA, respectively. All results are representative of at least three independent experiments and each value is the mean ± SD of three determinations. The results were reported as mean ± SD; *$p < 0.05$.

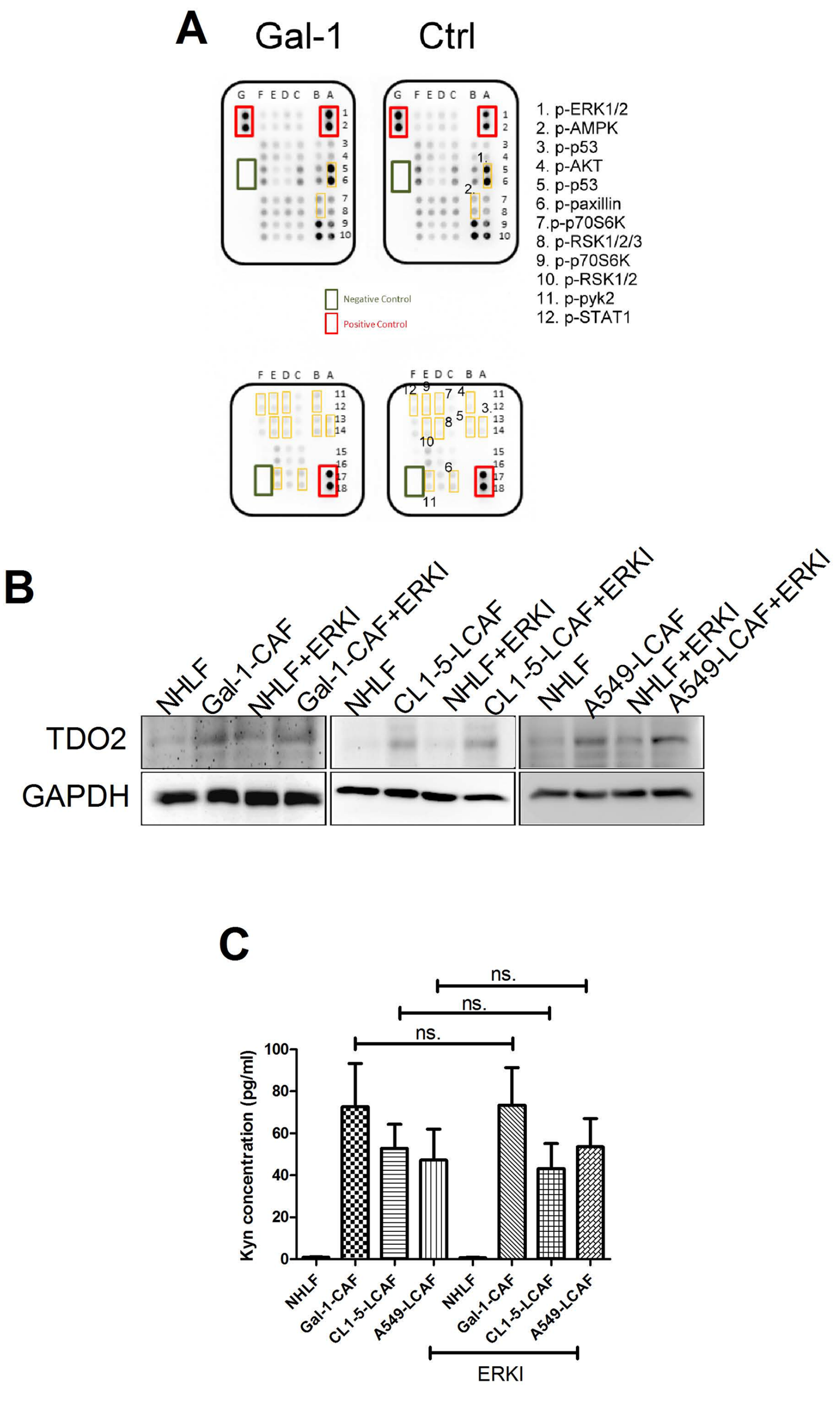

Supplementary Figure S9: The phosphorylated kinase profile of galectin-1-treated NHLF cells. A. rhgalectin-1 increased the phosphorylation of various kinase. The effect of ERK inhibitor on TDO2 upregulation **B.** and Kyn production **C.** NHLF cells were treated with rhgalectin-1 (1 µg/ml) for 3 h. The expressions of various kinases were assessed by Human Phospho-Kinase Array. NHLF cells were treated with ERK inhibitor for 1 h and then rhgalectin-1 for another 24 h. The expression of TDO2 and Kyn was assessed by Immunoblot and ELISA, respectively. All results are representative of at least three independent experiments and each value is the mean ± SD of three determinations. The results were reported as mean ± SD; *$p < 0.05$.
